# The Absence of Retroelement Activity Is Characteristic for Childhood Acute Leukemias and Adult Acute Lymphoblastic Leukemia

**DOI:** 10.3390/ijms23031756

**Published:** 2022-02-03

**Authors:** Shamil Urazbakhtin, Anastasia Smirnova, Anastasiya Volakhava, Elena Zerkalenkova, Maria Salyutina, Michael Doubek, Hana Jelinkova, Nelly Khudainazarova, Egor Volchkov, Laima Belyaeva, Ekaterina Komech, Sarka Pavlova, Yuri Lebedev, Karla Plevova, Yulia Olshanskaya, Alexander Komkov, Ilgar Mamedov

**Affiliations:** 1 Department of Genomics of Adaptive Immunity, Shemyakin-Ovchinnikov Institute of Bioorganic Chemistry, 117997 Moscow, Russia; shamil.urazbakhtin@gmail.com (S.U.); liliumanstist@gmail.com (A.S.); m.salyutina@gmail.com (M.S.); nelly_belly@bk.ru (N.K.); ekomech@gmail.com (E.K.); lebedev_yb@mx.ibch.ru (Y.L.); alexandrkomkov@yandex.ru (A.K.); 2 Center of Life Sciences, Skolkovo Institute of Science and Technology, 121205 Moscow, Russia; 3 Central European Institute of Technology, Masaryk University, 625 00 Brno, Czech Republic; volokhova_nastia@mail.ru (A.V.); Doubek.Michael@fnbrno.cz (M.D.); pavlova.sarka@fnbrno.cz (S.P.); Plevova.Karla@fnbrno.cz (K.P.); 4 Dmitry Rogachev National Medical and Research Center of Pediatric Hematology, Oncology and Immunology, 117997 Moscow, Russia; elena.zerkalenkova@fccho-moscow.ru (E.Z.); volchcov.egor@yandex.ru (E.V.); laimabel02@gmail.com (L.B.); yuliaolshanskaya@gmail.com (Y.O.); 5 Department of Internal Medicine, Hematology and Oncology, University Hospital Brno and Faculty of Medicine, Masaryk University, 625 00 Brno, Czech Republic; Jelinkova.Hana@fnbrno.cz; 6 Institute of Medical Genetics and Genomics, University Hospital Brno and Faculty of Medicine, Masaryk University, 625 00 Brno, Czech Republic; 7 Department of Molecular Technologies, Pirogov Russian National Research Medical University, 117997 Moscow, Russia

**Keywords:** acute leukemia, retroelements, mobile elements, tumor-specific insertions

## Abstract

Retroelements (RE) have been proposed as important players in cancerogenesis. Different cancer types are characterized by a different level of tumor-specific RE insertions. In previous studies, small cohorts of hematological malignancies, such as acute myeloid leukemia, multiple myeloma, and chronic lymphocytic leukemia have been characterized by a low level of RE insertional activity. Acute lymphoblastic leukemia (ALL) in adults and childhood acute leukemias have not been studied in this context. We performed a search for new RE insertions (Alu and L1) in 44 childhood ALL, 14 childhood acute myeloid leukemia, and 14 adult ALL samples using a highly sensitive NGS-based approach. First, we evaluated the method sensitivity revealing the 1% detection threshold for the proportion of cells with specific RE insertion. Following this result, we did not identify new tumor-specific RE insertions in the tested cohort of acute leukemia samples at the established level of sensitivity. Additionally, we analyzed the transcription levels of active L1 copies and found them increased. Thus, the increased transcription of active L1 copies is not sufficient for overt elevation of L1 retrotranspositional activity in leukemia.

## 1. Introduction

Retroelements (REs) occupy nearly a half of the human genome [1], still being predominantly inactive in normal cells. Their transcription, translation, and retrotransposition are repressed by various cellular mechanisms preventing the occurrence of new RE copies [2]. In addition, the vast majority of RE copies in the human genome are not capable of transcription and/or retrotransposition due to mutations and deletions in their regulatory regions and proteins as well as hypermethylation of their promoters. More than a hundred of retrotranspositionally-competent RE copies are present in the human genome and belong to the evolutionary “young” subfamilies of LINE1 (long interspersed nuclear elements; L1HS and L1PA2) that appeared in the genome 5 to 10 million years ago [3,4]. These autonomous retroelements are nearly 6 kb long and include two open reading frames (ORFs) coding for proteins that are necessary for their retrotransposition. The second class of retroelements—non-autonomous SINEs (short interspersed nuclear elements)—also include some potentially active retrotransposition-competent copies belonging to Alu (AluYa5, AluYb8, and some others) and SVA subfamilies [5]. These elements do not encode any proteins and use the LINE1 machinery for their retrotransposition [6].

The activation of retroelements happens under certain pathological conditions including schizophrenia and cancer [7,8]. Global DNA demethylation, chromatin remodeling, and changes in the expression of RE repressor proteins are believed to be major factors leading to RE activation in cancer cells [2]. RE derepression could result in the elevation of RE-derived transcripts and proteins in a cell, and the occurrence of new copies in various genomic locations. Interestingly, various cancer types are characterized by different levels of RE transpositional activity. For example, recent studies that are based on tumor whole-genome sequencing data [9,10] showed that epithelial tumors such as lung, colorectal, esophageal, and head and neck cancers have a high number of tumor-specific RE insertions (also called MEIs for mobile element insertions). The number of insertions may differ significantly from 1 to 10 to more than a hundred among samples of the same cancer type, whereas some samples do not carry new insertions at all. The majority of new RE insertions comprise of LINE1 elements [11] while novel Alu and SVA insertions are 100 times rarer than LINE1s. This phenomenon is explained by the preferential cis activity of LINE1 reverse transcriptase which, in most cases, integrates a cDNA copy of the RNA molecule that was a template for its translation [12]. Other cancer types, such as liver and kidney, are characterized by significantly lower RE insertional activity: only a few samples have 1 to 10 tumor-specific insertions. The same holds for blood cancers, although these types of malignancies have been studied to a lesser extent. The RE insertional activity was studied in patients with chronic lymphocytic leukemia (CLL), B-cell non-Hodgkin’s lymphoma (BNHL), myeloproliferative neoplasms (MPN), acute myeloid leukemia (AML), and multiple myeloma (MM) [9,13,14]. All these studies reported no or only a few tumor samples bearing new RE insertions. To the best of our knowledge, acute lymphoblastic leukemia (ALL) of both childhood and adult age, and childhood acute myeloid leukemia (AML) have not been studied for tumor-specific RE insertions.

New RE insertions can occur in any phase of tumor evolution, i.e., in premalignant cells, in tumor cells right after malignant transformation, or during tumor progression. Due to clonal proliferation, the former two types of insertions are present in the vast majority of tumor cells. In the case of ongoing RE activity during cancer progression, the new tumor-specific insertions can be present just in small subclones of malignant cells. Such subclones can potentially have a more aggressive or resistant phenotype and become dominant at relapse. Early-stage insertions can be detected in whole-genome sequencing (WGS) data with the use of specific computational pipelines [13,15,16]. Such pipelines identify and compare the coordinates of RE copies in matched tumor and normal sample pairs to reveal the tumor-specific insertions. In contrast, insertions that occur late during cancerogenesis may be present in a small portion of tumor cells and not reliably detectable by WGS with the standard coverage around 30×. It was estimated that WGS data can be used to identify tumor-specific RE insertions that are present in at least 25% of tumor cells [9]. Thus, the absence of detected retropositional activity in particular tumors, including blood cancer, can be a result of insufficient sensitivity of the methods that are used for its identification. More sensitive methods for RE insertion identification are based on enrichment for DNA fragments containing young and potentially active retroelement copies before high-throughput sequencing. This enrichment is based either on the amplification with an RE-specific primer [17,18,19] or on hybridization with RE-specific oligonucleotides and capture [20,21,22]. Although the targeted approaches are more sensitive, their significant drawback is the appearance of various artifacts in the course of library preparation that mimic true somatic RE insertions. Such artifacts are usually covered by few sequencing reads and can arise from a single molecular event, unlike insertions that are covered by multiple different reads in WGS data. For this reason, accurate verification of candidate insertion sequences by an orthogonal method is absolutely necessary [23].

Here we describe the first study of RE activity in adult and pediatric ALL and pediatric AML using the method that can reliably detect RE insertions in as little as 1% of tumor cells.

## 2. Results

### 2.1. Library Preparation, Sequencing, and Data Analysis

**Figure 1:**
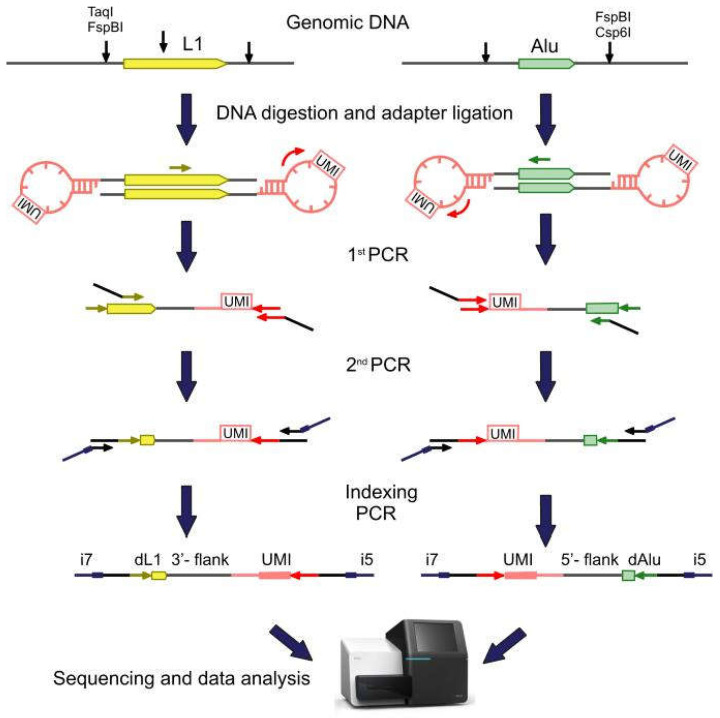
Principle of the method. gDNA was digested by either TaqI + FspBI (for L1), or FspBI + Csp6I (for Alu) and ligated to a stem-loop adapter (pink). Retroelement-specific primers (yellow and green arrows) were used for selective amplification of 3′ L1 or 5′ Alu flanking sequences (flanks). Indexing PCR introduced the sample barcodes and oligonucleotides that were necessary for Illumina sequencing (i5 and i7). dL1—part of an L1 element, dAlu—part of an Alu element, UMI—Unique Molecular Identifier.

In this study, we searched for tumor-specific insertions of the L1HS, AluYa5 (with AluYa8), and AluYb8 (with AluYb9) retrotransposon families in 14 childhood AML, 44 childhood ALL (31 B-cell and 13 T-cell), and 14 (10 B-cell and 4 T-cell) adult ALL paired samples. These subfamilies comprise 3 of 4 RE groups containing the most retropositioanally active elements. Tumor genomic DNA (gDNA) was isolated from bone marrow or peripheral blood with 23–99% (median 82%, all the samples except for three contained more than 50% of tumor cells) blasts in the disease onset, whereas normal gDNA of the same patient was obtained from either buccal swabs (in adult ALL cases) or bone marrow in complete (molecular MRD negative) remission (childhood ALL and AML). For library preparation, we used a modification of a previously published method [24,25]. The design of the method is shown in Figure 1. gDNA was digested by a mixture of selected endonucleases (FspBI and Csp6I for Alu, TaqI and FspBI for L1) to generate fragments that consisted of a retroelement (or its part, for L1s) and its adjacent genomic sequence (flank). The set of endonucleases was selected to obtain flanks of 25–800 bp long allowing their sequencing on Illumina machines and the correct mapping to the human reference genome. At the next step, the fragmented DNA was ligated to a stem-loop adapter containing unique molecular identifiers (UMI) [26]. These random 10 nt oligonucleotides were used to mark each DNA molecule containing an RE insertion with a unique barcode that was used to quantify the number of cells bearing each insertion. Next, primers that were specific to transpositionally-active RE subfamilies L1HS, AluYa5, and AluYb8 were used for the selective amplification of genomic flanks that were adjacent to each RE copy, i.e., 5′ flank in case of Alu, and 3′ flank in case of L1 (see Figure 1). These flanking sequences were used for the mapping of each insertion to the human reference genome after Illumina sequencing. A product of the first PCR was used in the second semi-nested PCR and finally an indexing PCR was carried out to introduce the sample barcodes and oligonucleotides that were necessary for Illumina sequencing.

We used a custom computational pipeline [27] to map all the sequenced insertions’ flanks to the human reference genome. The method specificity that was calculated as the percentage of target RE flanks among all sequences was 0.87–0.96 for AluYa5, 0.86–0.92 for AluYb8, and 0.83–0.98 for L1HS. The coordinates of the insertions in the paired tumor and normal samples were matched to identify the sample-specific insertions. Importantly, our pipeline enabled filtering out the majority of the artificial chimeric sequences that mimicked true RE insertions, including ligation and template-switch chimeras (see [27] for a detailed description of the computational pipeline and chimeric sequence filtration). Insertion was considered candidate tumor-specific when it was found in a tumor sample and was absent in the matched normal sample as well as in all other genomes from this and our previous studies. As an additional filtering criterion, we picked up only insertions that were marked by at least two UMIs. Such insertions were more reliable as the independent formation of chimeric molecules with an identical target sequence was very low. As a result, we identified 12 candidate tumor-specific L1 insertions and 36 candidate tumor-specific Alu insertions in all the samples. A total of 4 candidate L1 insertions were found in 3 out of 14 childhood AML samples, 6 insertions in 6 out of 44 childhood ALL samples, and 2 insertions in 2 out of 14 adult ALL samples. The distribution of candidate Alu insertions were as follows: 17 candidate tumor-specific AluYa5 or Yb8 insertions were found in 10 childhood AML samples, 18 insertions in 10 childhood ALL samples, and 1 insertion in one out of 14 adult ALL samples. All the candidate insertions were characterized by 2–3 UMI except one Alu having 6 UMI. In contrast, the reference Alu and L1HS insertions (i.e., present in the hg38 human genome or any of the human RE databases) were characterized by higher UMI numbers (average 209 for Alu and 174 for L1).

### 2.2. Validation of Candidate Tumor-Specific Insertions

**Figure 2:**
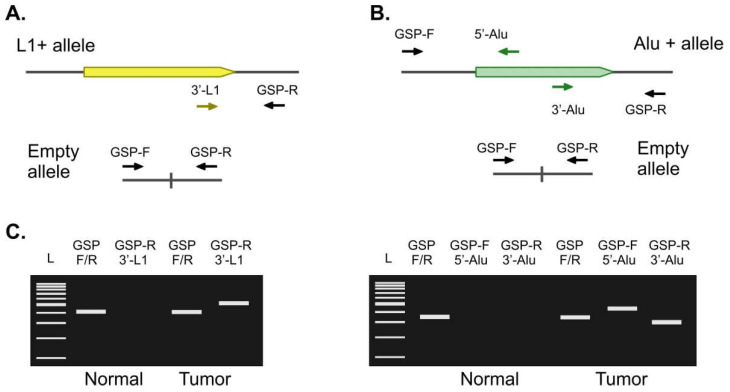
Validation of candidate tumor-specific insertions. (**A**). For L1, a 3′-L1-specific primer (yellow arrow) was used in combination with GSP-R primer (genomic locus-specific primer reverse, black) corresponding to the unique genomic 3′ flank of each L1 insertion. GSP-R and GSP-F primers were used to amplify an empty allele. (**B**). For Alu, an additional reaction with 5′-Alu-specific (green arrow) and GSP-F primers was performed. (**C**). The expected results of the PCR confirming tumor-specific insertion of L1 (**left**) or Alu (**right**).

Candidate somatic L1 insertions were validated by an independent method—locus-specific PCR (Figure 2A) with initial gDNA as a template. The primers that were complementary to the unique genomic 3′ flank of each L1 insertion (GSP-R—genomic locus-specific primer, reverse) were used in combination with a common universal primer annealing to the L1HS element (3′-L1 primer). A control reaction with the primers that were complementary to both 3′ and 5′ flank (GSP-F + GSP-R) was performed in parallel to amplify an insertion-free allele. Using this approach, we tested 10 out of 12 candidate L1 tumor-specific insertions. For the remaining 2 insertions, we were unable to design specific primers, as the candidate insertions were found inside other repetitive sequences. None of the candidate tumor-specific L1 insertions were validated by locus-specific PCR. A total of five insertions were found in both the tumor and normal samples. These insertions most probably represented the private germline or non-reference polymorphic insertions that were amplified by our method inefficiently. Thus, they were present in the tumor sample library at low counts (2–3 UMI) and absent in the corresponding normal sample library by chance. A total of five insertions were absent in both the tumor and normal samples. These candidates most probably represented the chimeric or incorrectly mapped sequences.

The same approach was used for the validation of the candidate Alu insertions. For Alu we performed 3 parallel PCR reactions using: (1) 5′-Alu-specific primer with 5′-flank primer (GSP-F + 5′-Alu); (2) 3′-Alu-specific primer with 3′-flank primer (GSP-R + 3′-Alu), and (3) 5′-flank primer with 3′-flank primer (GSP-F + GSP-R) as a control reaction (see Figure 2B). For the validation experiments, we selected 15 candidate Alu insertions that were found inside introns or in close proximity (5 kb upstream or downstream) of known human genes. Similar to L1, none of the candidate tumor-specific Alu insertions were validated by locus-specific PCR. A total of five insertions were found in both the tumor and normal samples, whereas 10 were absent in both samples.

### 2.3. Evaluation of the Method Sensitivity

The methods that were based on targeted sequencing of RE insertion sites are, by default, more sensitive than WGS allowing for detection of insertions that are present in a small portion of cells. However, the exact sensitivity of the commonly used methods in this respect is unknown. Recently, we directly measured the sensitivity of our selective amplification-based approach for somatic Alu insertions identification [24]. For this purpose, we mixed cells from four healthy individuals in different proportions (ranging from 1:100 to 1:1000), prepared libraries containing Alu flanks, sequenced them, and used polymorphic Alu insertions that were present in individual genomes to evaluate the potential of the method to detect insertions that are present in 0.1–1% of cells in a sample. We showed that with the sequencing depth of 1 million reads per sample, the majority (80%) of insertions that are present in 1% of cells is reliably detected in the sequencing data.

Here we repeated this experiment for the L1 insertions. We isolated gDNA from peripheral blood mononuclear cells of two healthy individuals, prepared L1 3′-flanking libraries in the same way as for the paired tumor/normal samples, sequenced them, and identified 12 known polymorphic L1HS insertions that were present in the genome of individual 1 and were absent in the genome of individual 2. Then, we isolated and mixed T cells from these two volunteers in the proportion of 1:100 (individual 1:individual 2) using FACS in two replicates. The prepared library was sequenced with the sequencing depth that corresponded to the average number of sequencing reads that were obtained for tumor and normal samples, i.e., approximately 500,000 reads. In the obtained sequencing dataset, we searched for the 12 polymorphic insertions that were present exclusively in the genome of individual 1 (i.e., in 1% of cells) and found 7 and 8 of them in the replicates 1 and 2, respectively. An insertion was considered to be detected when it was covered by two or more different UMIs. Of the 12 insertions, six (50%) were found in both replicates. Thus, we conclude that our method is capable of detecting approximately a half of L1HS insertions present in 1% of cells.

### 2.4. Transcription of Active L1 Retroelements in Leukemia

**Figure 3:**
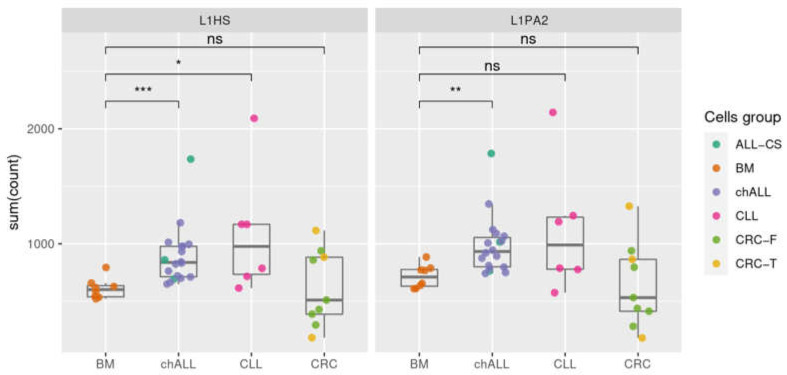
Transcription of active L1 copies in leukemia, colorectal cancer, and normal bone marrow samples. Each dot indicates the number of RNA-Seq reads that were mapped to the potentially traspositionally-competent L1HS (**left**) or L1PA2 (**right**) from L1base. BM—normal bone marrow, chALL—childhood acute lymphoblastic leukemia, ALL-CS—chALL samples from the present study, CLL—chronic lymphocytic leukemia, CRC—colorectal cancer with (CRC-T) or without (CRC-F) new tumor-specific insertions. Adjusted *p*-value (Padj), * *p* < 0.05, ** *p* < 0.01, *** *p* < 0.001, ns—non-significant.

The activation of RE in cancer depends on multiple molecular mechanisms including methylation, chromatin modification, transcriptional activation, and transport of the RE ribonucleoprotein complex into the nucleus. Which scenario contributes to the RE activation in a particular cancer type is poorly understood. However, one of the key indications of possible RE activation could be the increment of transpositionally-competent L1 RNA. Only these RNAs can produce active ORF2 protein that is necessary for reverse transcription and genomic insertion of a new L1 or Alu copy. To evaluate the bulk transcription level of transpositionally-competent L1s, we performed RNA-Seq for 3 leukemia samples that were analyzed for RE insertions in this study. We also used RNA-Seq data that was generated in our lab for 15 childhood ALL and 9 colorectal cancer samples. In 3 out of 9 colorectal cancer samples, we previously identified tumor-specific L1 insertions using our method [24]. Additionally, we downloaded 7 normal bone marrow [28] and 6 chronic lymphocytic leukemia [29] whole-transcriptome data that were generated using the same approach (i.e., RNA-Seq of polyA fraction; the accession numbers GSE162427 and E-MTAB-1733, respectively). We down-sampled all the datasets to 4.5 million reads (smallest sample) and mapped the reads to the reference database L1base that includes transpositionally-competent L1HS and L1PA2 [4]. We counted only those reads that mapped to the reference with 100% identity to exclude the majority of transcripts originating from other transpositionally-inactive L1s. In general, we observed a high variation in the levels of active L1 transcription among the tumor samples of any type, whereas normal bone marrow samples showed uniform L1 transcription levels distribution (Figure 3). A statistically significant increase in the transcription of potentially active L1 copies was observed in childhood ALL compared to normal bone marrow for both L1HS and L1PA2 elements (*p* = 0.00026 and *p* = 0.003936, respectively; Mann–Whitney test, with Benjamini–Hochberg correction). Interestingly, the colorectal tumors both with and without proven tumor-specific L1 insertions did not show significant elevation of active L1 transcripts compared to either the normal bone marrow or leukemia samples. These results indicate that transcription of active L1 copies is not sufficient for the effective transposition process and is dependent on other factors.

## 3. Discussion

Retropositional activity can play a significant role in tumor initiation and clonal evolution. Alterations in gene expression or large genomic rearrangements that are due to new RE insertions can promote malignant transformation. Examples of tumor initiation that are likely mediated by REs come from several studies [13,20,30,31] with the most convincing ones observed in colorectal cancer where independent reports of insertions in the well-known tumor driver APC gene were found in several patients [30,31]. The latter study by Cajuso et al. also reported the correlation between the presence of new tumor-specific RE insertions and poor disease prognosis. New insertions in transformed tumor cells can promote clonal evolution affecting the tumor cell phenotype, making them more invasive, faster dividing, and hypothetically less sensitive to the immune response. On the other hand, the activity of REs and genes that are involved in RE activation during tumor progression can play an opposite role. For example, the inactivation of H3K9 methyltransferase SETDB1 in AML leads to the activation of RE which, in turn, stimulates the type I interferon response and apoptosis [32]. In another example, the downregulation of L1 expression can increase tumor cell proliferation in colorectal cancer [33]. It can be speculated that although the activity of REs in initial cancerogenesis could be beneficial, the silencing of RE activity in tumor cells during progression can be a substantial advantage in clonal evolution or even the matter of tumor cell survival. Thus, the identification and monitoring of the transpositional activity in the tumor genome is of significant importance in the understanding of cancer evolution. Depending on the cancer stage at which the REs were activated, the portion of cells bearing particular insertions is different. “Early” insertions are present in most or a significant portion of cancer cells, whereas “late” insertions are found in a small percentage of cells. Many large-scale RE activity studies in cancer were based on WGS data allowing only the detection of “early” insertions. These studies reported a high level of transpositional activity in some cancer types and very low or absent activity in other malignancies. Hematological disorders belong to the latter category, however, comprehensive studies with highly sensitive methods have not been performed. Childhood leukemia that is characterized by different pathogenesis and disease course compared to adult leukemia has not been studied at all. Similarly, no studies exploring tumor-specific RE insertions in adult lymphoblastic leukemia have been published to date. In the present study, we aimed to close this gap by the identification of RE insertions in a large cohort of patients with acute leukemias using a sensitive method that enables the detection of Alu and L1 insertions that are present in as little as 1% of tumor cells. Our results indicate that RE transposition is not activated in childhood and adult leukemia neither at the disease initiation nor in the later stages of tumor development. These results are in good agreement with previously published studies. Similarly to pediatric AML that was analyzed in this study, no tumor-specific insertions were found in 18 adult AML samples using the WGS approach [13]. Taken all together, our and previous data allow drawing an overall conclusion that, in contrast to many other cancer types, RE activity is not characteristic either for adult or for pediatric leukemias. The reason for different RE activity in various cancer types is poorly understood. One of the possible explanations is the activation of different gene pathways in various cancer types. In normal cells, retroelements are repressed by multiple levels of cellular mechanisms and virtually all of them have to be broken to activate RE transposition. For example, transcriptional activation is not enough to produce new insertions. Another possible hypothesis is that the studied leukemia types have a low tolerance to RE insertional activity in contrast to other cancer types. RE activation in pre-leukemic cells could result in extensive cell death and only those subclones that kept REs under stringent control could survive and develop leukemia. The activation of RE can lead to leukemic cell elimination during cancerogenesis or especially in course of chemotherapy, when substantial cellular modifications occur. This assumption could be confirmed by implementing alternative therapeutic approaches that are based on the modulation of RE activity. Another way to prove this hypothesis is to change the methodological approach for RE insertion studies. The current standard for the detection of cancer-related RE activity is to analyze DNA/RNA from living cells rather than dead ones. In other words, the default assumption is that tumor-specific insertions are either beneficial or neutral for the malignant cell. We believe that the inclusion of genomic material that originates from dead cancer cells into the analysis, for example cell-free DNA or nuclear DNA from apoptotic cells, could provide novel insights into the RE activity in cancer. RE insertion profiles of this kind of DNA and DNA from living cancer as well as corresponding normal cells should be compared to reveal the complex structure of RE/cancer-cell relationships, not only in leukemia but also in all previously analyzed cancer types.

## 4. Materials and Methods

### 4.1. Sample Collection and DNA Isolation

The study was approved by the local institutional ethics committees and all the patients or their legal representatives (parents) and also the two healthy donors provided informed consent with the use of their samples and data for research purposes. The patient cohort characteristics is summarized in Table 1. The individual patient descriptions are given in Table S1.

In pediatric samples, bone marrow aspirates that were obtained at diagnosis were subjected to conventional cytogenetics [34], FISH and RT-PCR [35] to search for AL-associated recurrent chromosomal aberrations. The KMT2A gene rearrangements were further characterized by LDI-PCR [36] or NGS [37,38]. The respective chromosomal rearrangements were used for PCR-based MRD monitoring [35,39] and MRD-negative remission samples were used as normal controls. For adult ALL samples, mononuclear cells were obtained from BM and PB using gradient centrifugation on the Ficoll-Paque PLUS medium (GE Healthcare, Waukesha, WI, USA). gDNA from BM and PB was extracted with QIAamp DNA Mini Kit (Qiagen, Venlo, The Netherlands) according to the manufacturer’s protocol. gDNA from buccal swabs that were used as normal controls in the adult ALL samples was isolated using the MGT-02 401 MagCore Genomic DNA Tissue Kit (RBC Bioscience Corp., New Taipei City, Taiwan) on the MagCore Automatic Isolator following the instructions of the manufacturer.

### 4.2. Library Preparation and Sequencing

For the Alu flanks library preparation, 30 ng of gDNA was digested in 10 μL of 1× FD buffer with 5U of FspBI and 5U of Csp6I (all Thermo Fisher Scientific, Waltham, MA, USA) for 30 min at 37 °C. For adapter ligation, fragmented DNA was diluted in 20 μL of 1× FD Buffer with 20 µmol of ATP (Thermo Fisher Scientific), 50 pmol of stem-loop (SL) adapter (St19N10hook_v2) (see Table S2 for oligonucleotide sequences), 10U of T4 DNA ligase (Thermo Fisher Scientific), and incubated at 22 °C for 30 min. Next, 50 pmol of anti-adapter (antiHook-TA), additional 5U of FspBI and Csp6I endonucleases were added and the mixture was incubated at 22 °C for 30 min and 37 °C for additional 30 min. AntiSL-adapter inactivates SL-adapters, and endonucleases decrease the number of ligated chimeric molecules. The ligation reaction product was purified with 0.8 V of AmPure XP beads (Beckman Coulter, Brea, CA, USA), split, and used in two parallel 1st PCR reactions for Alu Ya5 and Alu Yb8 (25 µL each) containing 1× Encyclo Buffer, 1× Encyclo polymerase, 200 µM of each dNTP (all Evrogen, Moscow, Russia), 0.2 µM of 5′-flank oriented AluYa5 or AluYb8-specific primer, and primer St19okor corresponding to the part of SL-adapter before the UMI. The amplification profile was: 95 °C for 2 min, followed by 12 cycles of 20 s at 95 °C, 20 s at 65 °C, and 1 min at 72 °C with ramp rate 1 °C/s. A total of 2 µL of the obtained 1st PCR product from each Ya5 and Yb8 reactions was added to two parallel 2nd PCR reactions containing 200 µM of each of dNTP, 0.2 µM of the Alu-specific primer (common for Ya5 and Yb8, corresponding to very 5′ part of the Alu), adapter-specific primer korNxtSt19okor, and 1× Encyclo polymerase in 1× Encyclo Buffer. The amplification profile was: 2 min at 95 °C followed by 10 cycles of 20 s at 95 °C, 20 s at 60 °C, and 1 min at 72 °C. A total of 2 µL of the 2nd PCR product was used in the third indexing PCR 25 µL reaction containing 200 µM of each dNTP, 0.2 µM of each Nextera Indexing primers and 1× Encyclo polymerase in 1× Encyclo Buffer amplified for 12 cycles using the same amplification profile as for the 2nd PCR. The indexing PCR products were purified with AmPure XP beads and mixed equimolarly for sequencing.

L1HS library preparation was performed in the same way with the following modifications: (1) The restriction mixture was prepared with 5U of TaqI instead of Csp6I and incubated for 30 min at 37 °C followed by for 30 min at 65 °C; (2) the fragmented DNA was ligated with two SL-adapters (St19N10hook_v2 and St19N10hook_TaqI) because the restriction enzymes that were used produce different 5′ overhangs. Accordingly, two anti-adapters (antiHook-TA and antiHook-TaqI) were added at the second ligation step; and (3) the 1st and 2nd PCR were carried out with L1HS-specific primers (towards 3′ part of L1) and modified amplification profiles: 95 °C for 2 min, followed by 14 cycles of 20 s at 95 °C, 20 s at 58 °C and 1 min at 72 °C for the 1st PCR, and 95 °C for 2 min, followed by 12 cycles of 20 s at 95 °C, 20 s at 65 °C, and 1 min at 72 °C for second and indexing PCRs.

Libraries were sequenced on Illumina NextSeq or MiSeq (Illumina Inc, san Diego, CA, USA), paired-end 150 + 150. For the read numbers and other library characteristics see Table S3. Following data processing and tumor-specific insertion identification were performed as described in [27].

### 4.3. Validation of RE Insertions by Locus-Specific PCR

iDOP PCR (improved degenerate oligonucleotide-primed PCR) [40] was used to pre-amplify gDNA of tumor and normal samples. A total of 30 ng of gDNA was amplified in 25 µL PCR reaction mixture containing 1× SD buffer (Mg-free), 1× SD polymerase, 3 mM MgCl_2_, 240 µM of each of dNTP, and 0.2 µM of the iDOP primer (5′-GTGAGTGATGGTAGTGTGGAGNNNNNNATGTGG -3′) (all Evrogen, Russia). The amplification profile was: 92 °C for 2 min, followed by 6 cycles of 1 min at 92 °C, 1 min at 30 °C, 3 min at 68 °C followed by 14 cycles of 30 s at 92 °C, 30 s at 62 °C, 3 min at 68 °C, and a final extension 2 min at 68 °C.

The primers for the specific genomic regions (see Table S4) were designed with the use of primer-blast [41] and GeneRunner programs. 25 µL PCR reactions containing 200 µM of each of dNTP, 0.2 µM forward and reverse primers each, and 1× Encyclo polymerase in 1× Encyclo Buffer. The amplification profile was as follows: 2 min at 94 °C followed by 35 cycles of 20 s at 94 °C, 20 s at 60 °C, and 1 min at 72 °C.

### 4.4. Evaluation of Method Sensitivity

Peripheral blood mononuclear cells (PBMC) of two healthy donors were isolated from peripheral blood by a standard Ficoll-Paque (PanEco, Moscow, Russia) centrifugation protocol. Polymorphic L1 insertions that were specific for the donors were identified as described previously [24]. Briefly, we isolated the gDNA from PBMC of individual 1 and individual 2, prepared L1 flank libraries separately without mixing the cells, and sequenced them (MiSeq 2 × 150 233,390 reads for individual 1 and 170,120 for individual 2). We processed the raw sequencing reads to obtain a metatable containing a list of the identified L1 insertions in the two individuals with their genomic positions and read counts for each. Based on the read counts for the known fixed L1HS insertions, we identified the value of the first quartile (Q1) in each individual dataset. Then, using the metatable, we found polymorphic insertions that had a read count ≥Q1 in the individual 1 and 0 reads in the individual 2. We identified 12 such insertions that were present in the genome of individual 1 and absent in the genome of individual 2. Then, we mixed the T-cells of individuals 1 and 2 using FACS. The cells of individual 2 were not stained, the cells from individual 1 were stained by the CD3-eFluor450 (UCHT1, eBioscience, San Diego, CA, USA) antibody. FACS sorting was performed in 5 replicates on BD FACS Aria III (BD Biosciences, Franklin Lakes, NJ, USA). The cells were gated based on side and forward scatter. For each cell mixture replicate we first sorted 50,000 cells of individual 2 and then 500 cells of individual 1. After sorting, three out of five aliquots were resorted to evaluate the resulting number of cells in the mixture. gDNA from the sorted cells (2 aliquots) was isolated using QIAGEN DNeasy Blood and Tissue Kit (Qiagen, Venlo, The Netherlands). The sequencing libraries were prepared and sequenced on Illumina MiSeq 2 × 150. Sequences of the flanks of the 12 insertions that were present in the genome of individual 1 and absent in the genome of individual 2 were searched in the raw sequencing reads, allowing for 1 mismatch/indel. The insertion was considered “found” if it had at least 2 sequencing reads corresponding to its flank in the dataset.

### 4.5. Transcriptome Sequencing and Data Analysis

RNA was isolated from fresh-frozen cells with Trizol reagent (ThermoFisher Scientific) according to the manufacturer’s protocol. RNA-seq library preparation was performed with NEBNext Ultra II Directional RNA Library Prep Kit for Illumina (New England Biolabs, Ipswich, MA, USA). The libraries were sequenced on Illumina NextSeq 550 (paired-end 75 + 75) generating from 17,805,150 to 18,351,291 sequencing reads per sample. In addition to three samples that were tested for new RE insertions in the present study, we used datasets that were obtained in other studies including 7 normal bone marrow [28] and 6 chronic lymphocytic leukemia [29] (accession number GSE162427 and E-MTAB-1733, respectively). Raw sequencing reads were processed with Cutadapt (https://doi.org/10.14806/ej.17.1.200, accessed on 10 January 2022) to remove adapters and shorten reads to 75 + 75 nt to match the read length of our libraries. PRINSEQ [42] was used to remove PCR duplicates and the low quality reads (parameters used: -min_qual_mean 20, -derep 1, -ns_max_p 3). Each dataset was split into a number of batches of 4,500,000 reads each with split2 option of SeqKit [43]. The reads were aligned using Magic-BLAST [44] with parameter -limit_lookup F on sequences of active L1HS (151 elements) and L1PA2 (85 elements) insertions that were extracted from L1base2 [4] which contained full-length L1s with intact ORF2. A bulk transcription level of such potentially active L1HS and L1PA2 was calculated as a sum of all the unique proper paired reads which aligned exactly to at least 1 reference L1 sequence from L1base2. Data analysis was performed in R (https://www.R-project.org/, accessed on 10 January 2022) using the ggplot2 (https://ggplot2.tidyverse.org, accessed on 10 January 2022) package for visualization.

## Figures and Tables

**Table 1:** Patient cohorts’ description.

Diagnosis	Number	Age (Median)	Gender (Male/Female)	Percent of Malignant Cells in the Tumor Sample (Median)
Pediatric T-ALL	13	1–15 (10)	8/5	41–95 (87.5)
Pediatric B-ALL	31	1–16 (4)	16/15	42–86 (77.5)
Pediatric AML	14	1–15 (7)	6/8	55–90 (74)
Adult T-ALL	4	29–70 (54)	2/2	75–96 (86)
Adult B-ALL	10	19–51 (26)	7/3	23–86 (72)

## Data Availability

Raw sequencing data are available at https://www.ncbi.nlm.nih.gov/bioproject/PRJNA800807, accessed on 10 January 2022.

## Supplementary Materials

The supporting information can be downloaded at: https://www.mdpi.com/article/10.3390/ijms23031756/s1.
