# Supplementary material for: The Absence of Retroelement Activity Is Characteristic for Childhood Acute Leukemias and Adult Acute Lymphoblastic Leukemia
Source: Int J Mol Sci. 2022 Feb 3;23(3):1756. doi: 10.3390/ijms23031756 (PMC8835895; doi:10.3390/ijms23031756)
Supplement: Supplementary file 1 [file ijms-23-01756-s001.zip › Table S2.pdf]

Table S2. Oligonucleotides used for library preparation.

Name	Sequence	Description
**Adapter ligation**		
St19N10hook_v2	TAAGCACGACGCCTGCGTGCTGCGGNNNNNNNNNNAGGCG TCGTGCT	SL-Adapter
St19N10hook_TaqI	CGTGCACGACGCCTGCGTGCTGCGGNNNNNNNNNNAGGCG TCGTGCA	SL-Adapter
antiHook-TA	pTAGTGTGCTCGTAGTCAAAAGACTACGAGCACAC	AntiSL-adapter
antiHook-TaqI	pCGGTGTGCTCGTAGTCAAAAGACTACGAGCACAC	AntiSL-adapter
**1st PCR**		
AY107	TCACCGTTTTAGCCGGGA	AluYa5-specific primer
BY267	GAGACCATCCCGGCTAAAAC	AluYb8-specific primer
St19roko-L1HS	CGTCGTGCGTAGATGACAC	L1HS-specific primer
St19okor	GCGTGCTGCGG	Adapter-specific primer
**2nd PCR**		
korNxtSt19ok	TCGGCAGCGTCAGATGTGTATAAGAGACAGCGTGCTGCGG	Adapter-specific primer
korNxtAY19	CGTGGGCTCGGAGATGTGTATAAGAGACAGAGCCACCGCGC	AluY-specific primer
KorNxt-L1HS	CGTGGGCTCGGAGATGTGTATAAGAGACAGCATGTACCCTAA AACTTAGA	L1HS-specific primer
