# Supplementary material for: The Absence of Retroelement Activity Is Characteristic for Childhood Acute Leukemias and Adult Acute Lymphoblastic Leukemia
Source: Int J Mol Sci. 2022 Feb 3;23(3):1756. doi: 10.3390/ijms23031756 (PMC8835895; doi:10.3390/ijms23031756)
Supplement: Supplementary file 1 [file ijms-23-01756-s001.zip › Table S4.pdf]

Table S4. Primers used for insertion validation.

Name	Sequence
**Genomic locus specific primers for L1HS (GSP)**	
61-AML-c2_For	CCAGAGTGCCTGTTTCCCTA
61-AML-c2_Rev	ATTAAGGCACCAATAACTCAA
10-BALL-c15_For	GCCTGAGTTAGTTTTTGTATAAGA
10-BALL-c15_Rev	AAGATGCAAAGACAACTCAATGA
32-BALL-c1_For	TTTGAATGAATTAGAAAAGAGGTC
32-BALL-c1_Rev	GAGCACATAGACAGAGGAAG
38-TALL-c3_For	TCCTGACAGCCTCATTGTAAA
38-TALL-c3_Rev	CAAGACTTTGGTTCCATTCCC
41-TALL-cX_For	CCAAATGCCAGGCCTGTAAT
41-TALL-cX_Rev	AAGTGTGACCAAACACATGCC
45-TALL-c15_For	AATGTAATCACCTTACCCAATG
45-TALL-c15_Rev	CAGCCCCTTTTCAGTGTTTC
ALLB0069_L1_chr8_F	GATGGTTAACATTGGGAAGCC
ALLB0069_L1_chr8_R	CCCTCTTTCTTCATATCTTCAC
ALLB0101_L1_CHR22_F	ATAGACAGCAAATAAAATGTGAAC
ALLB0101_L1_CHR22_R	GTGTATGAGTGAGTGTGAGTG
**Genomic locus specific primers for AluYa5/Yb8**	
50-AML-c9_For	ACCTGACTCTGCACCTTTGA
50-AML-c9_Rev	TGGGAGCCCTGCTGTCCT
50-AML-c13_For	CCGGTGGAGAGAAGGGGATA
50-AML-c13_Rev	AATACTGAACAGGTGTTTGGCA
51-AML-c10_For	TTTAGTCTAACGAATAGGTGCT
51-AML-c10_Rev	ACACAAAATAATTAAAGATAAGTCA
52-AML-c2_For	CTGCAGGCTTCATGTCCTAA
52-AML-c2_Rev	GAGACTGTGGAAAAAGAACTC
58-AML-c6_For	TGTTGCAGCCTTGTGGGTG
58-AML-c6_Rev	GAAAATCATTGGGATGATGGGA
58-AML-c16_For	CCTTCACATTCATGACAACTTC
58-AML-c16_Rev	GACTGGGGAGCCACAGGG
60-AML-c9_For	CACTTTGTAATGTAAAATGGGAT
60-AML-c9_Rev	GCCGGGTGCGATGGCTC
63-AML-c11_For	GGAGGCAGAGAGGCAGAG
63-AML-c11_Rev	ATCAGGGTGTGGAGGATGTC
33-BALL-c15_For	GTCTTGGAGTGCATGGAAATA
33-BALL-c15_Rev	GGCTCCCTCTTGGTCTCTT
35-TALL-c3_For	GGTCTTACTCAGAGGAAGGA
35-TALL-c3_Rev	GGATTACAGGAAGCAGTCATC
39-TALL-c19_For	AGTCCTTGTGTGTTTTAGATAC
39-TALL-c19_Rev	ACCAATCCCCTGAGAATACC
39-TALL-cx_For	CACCACACTGAACAAATGCTA
39-TALL-cx_Rev	ACTCCACAGGATGGAATGAAG
42-TALL-c8_For	GAACTCTGGCTGATGGCAAA
42-TALL-c8_Rev	CCTACCTCTCCACACCCTC
43-TALL-cX_For	GCACAAGATGCGAGGTAGGT
43-TALL-cX_Rev	CAAGGATGGATAGGTGGGC
ALLB0124_Alu_chr4_F	CTGCATTGCCCAGAACATTAT
ALLB0124_Alu_chr4_R	GATGCCTTAGACTATACCGTG

Primers annealing to retroelements	
L1HS specific primer (3’-end proximal)	
b3-L1HS For2	TGGGAGATATACCTAATGCTAGATGACAC
AluYa5 specific primers (5’- and 3’-end proximal)	
bAY102For	GAGACCATCCCGGCTAAAAC
bAY107Rev	TCACCGTTTTAGCCGGGA
AluYb8 specific primers (5’- and 3’-end proximal)	
b-BY270F	CCACTGCAGTCCGCAGTCCGG
b-BY267R	CCCAGGCCGGACTGCGGAC
